# Supplementary figures and images for: Safety of outpatient vs. inpatient anterior cervical discectomy and fusion: a systematic review and meta-analysis
Source: PeerJ. 2025 Sep 22;13:e20045. doi: 10.7717/peerj.20045 (PMC12462687; doi:10.7717/peerj.20045)

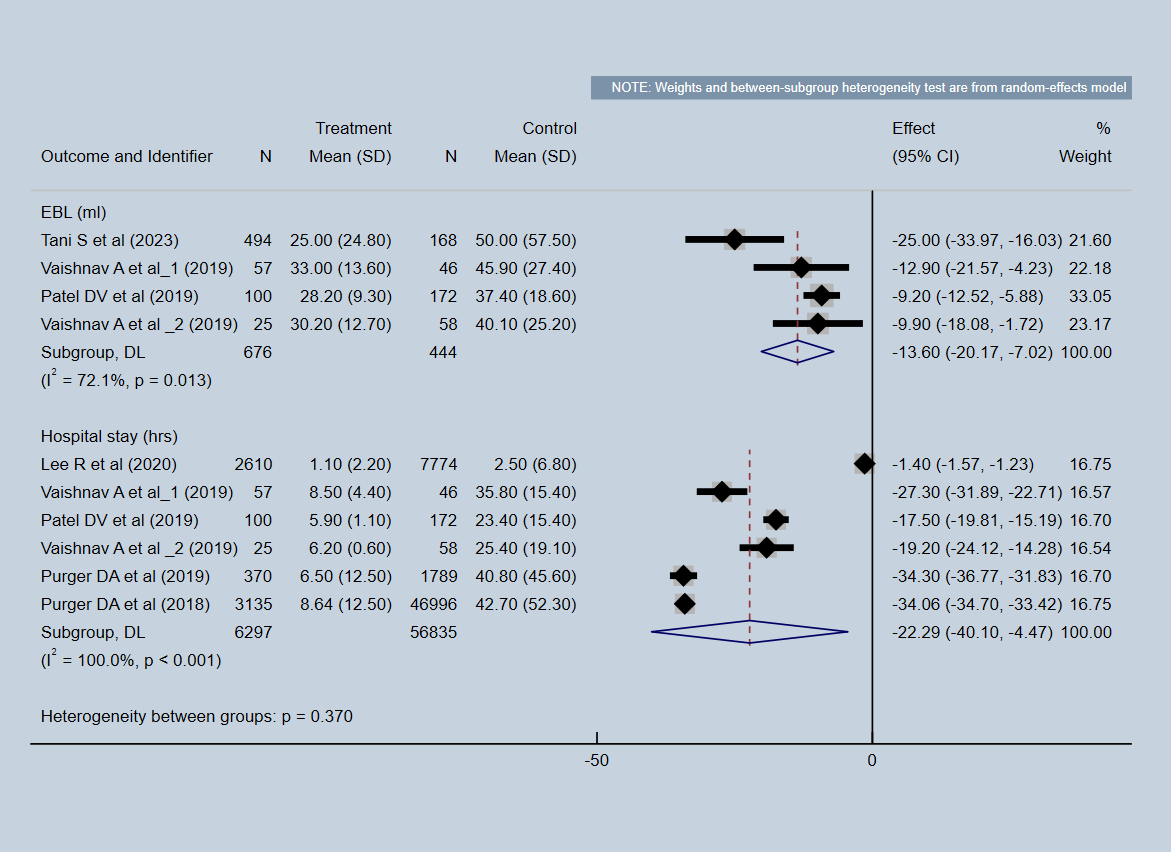

Supplement: Supplemental Information 1 [file peerj-13-20045-s001.tif]

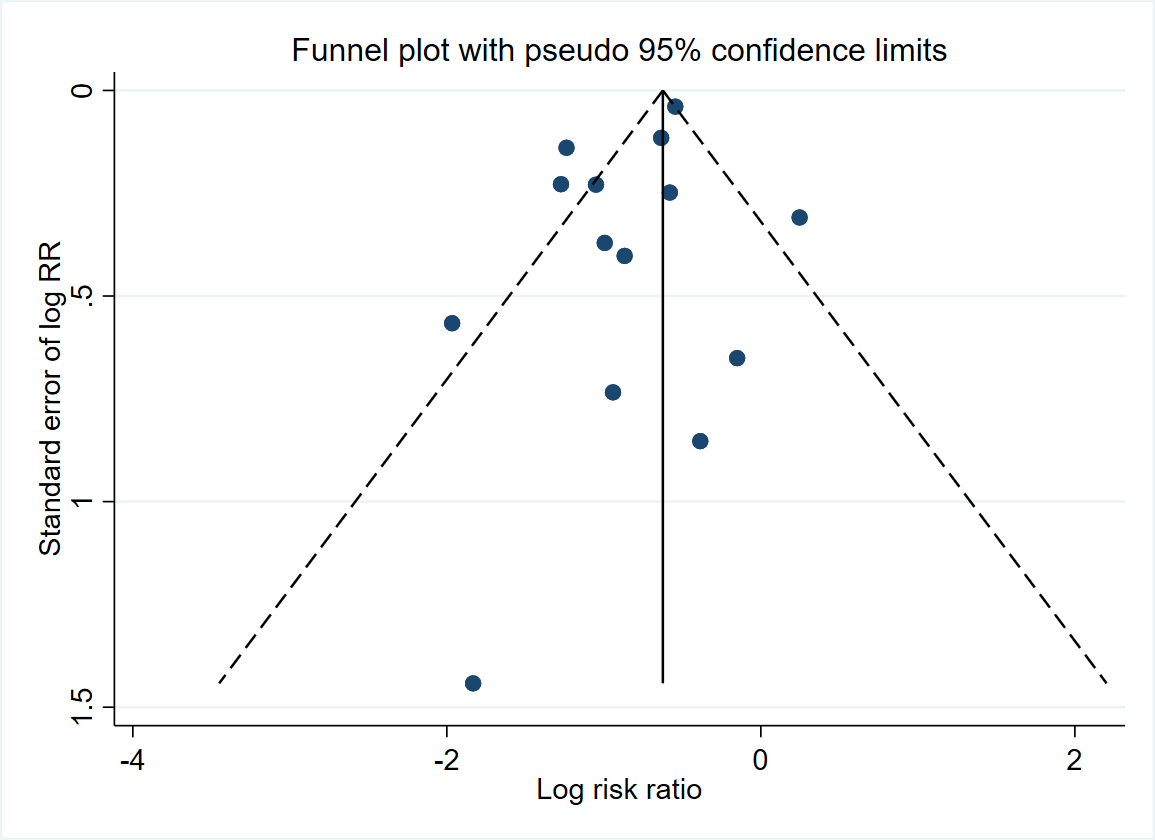

Supplement: Supplemental Information 2 [file peerj-13-20045-s002.tif]

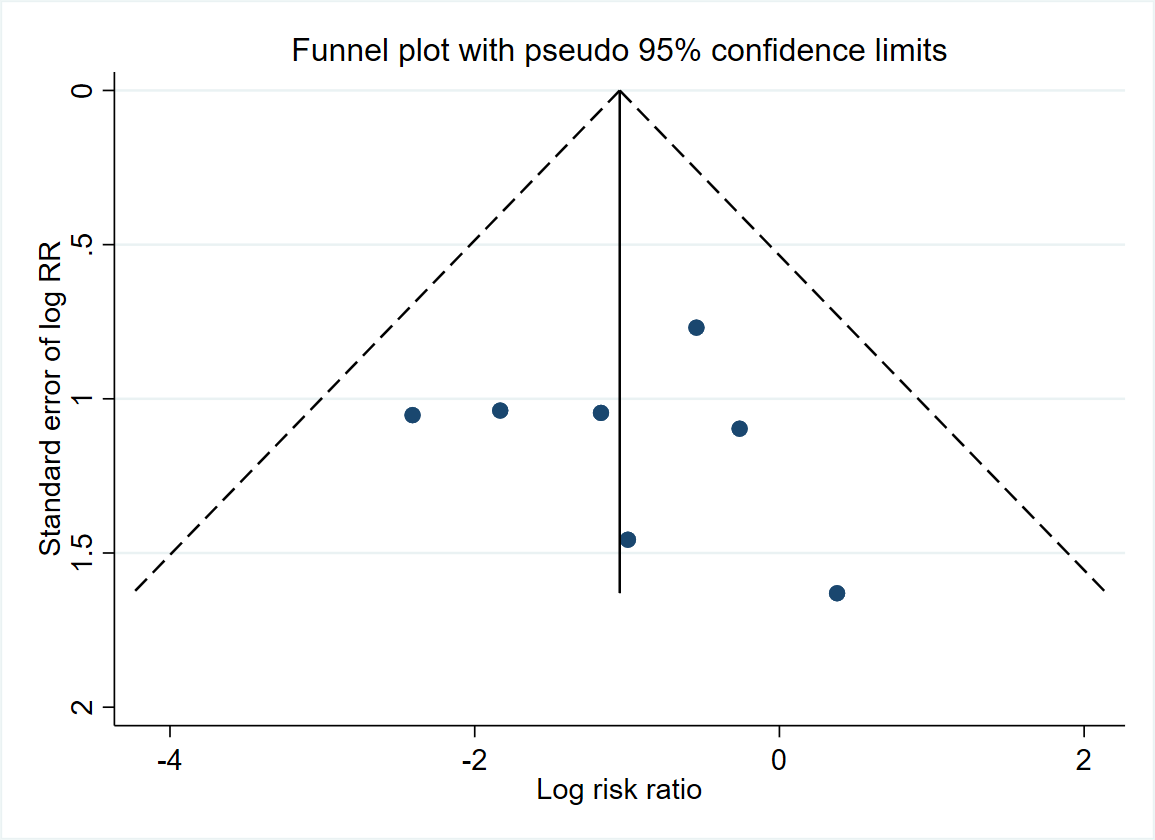

Supplement: Supplemental Information 3 [file peerj-13-20045-s003.tif]

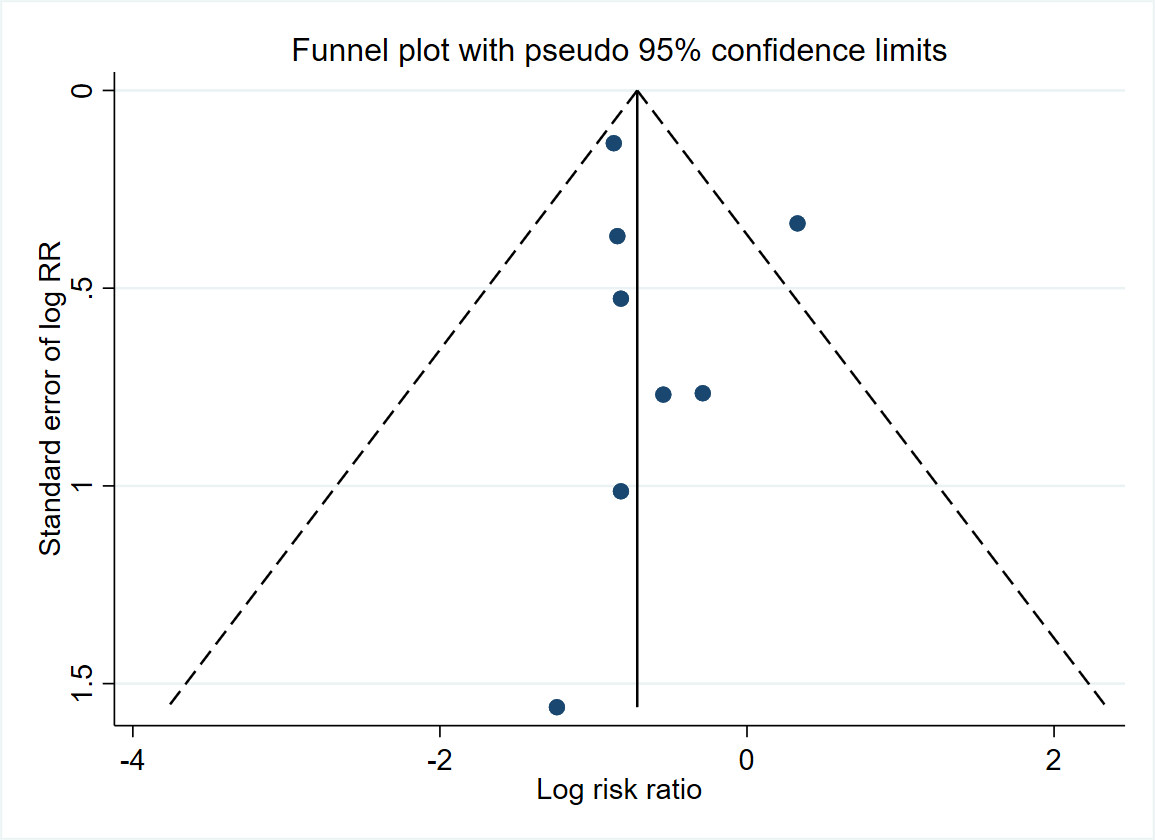

Supplement: Supplemental Information 4 [file peerj-13-20045-s004.tif]

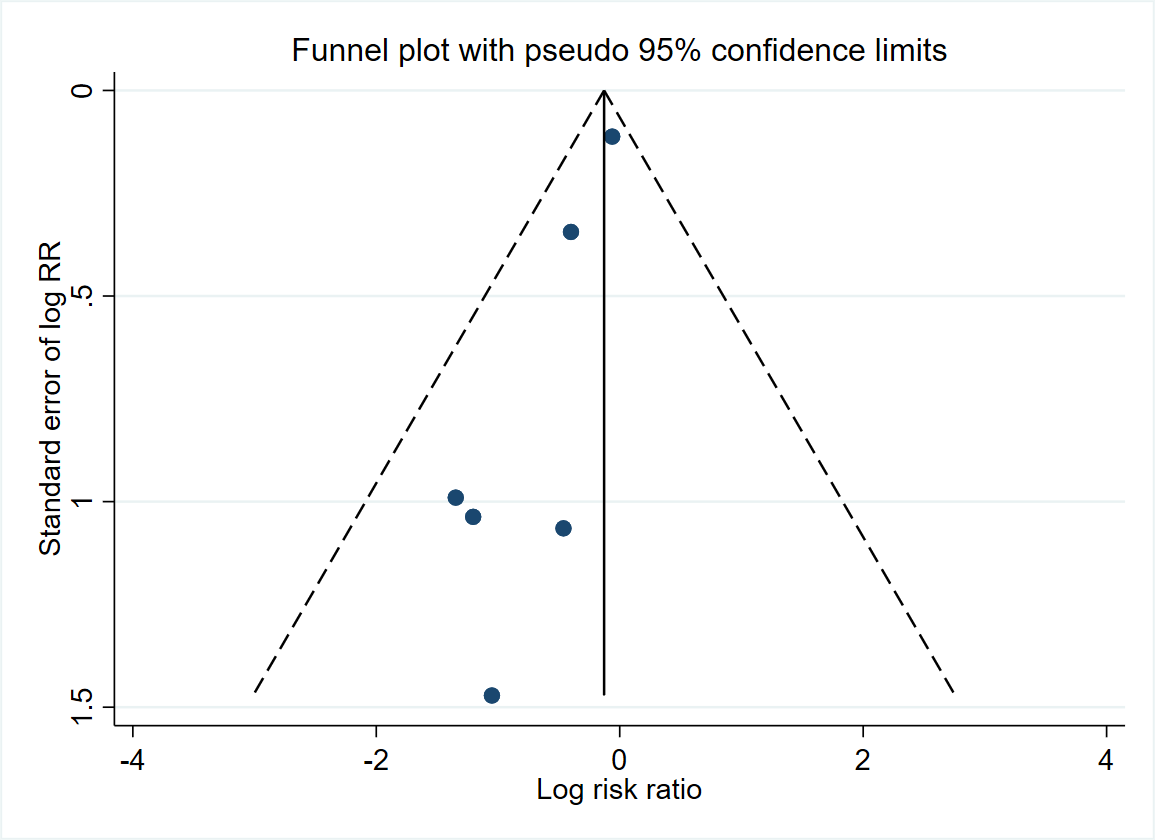

Supplement: Supplemental Information 5 [file peerj-13-20045-s005.tif]

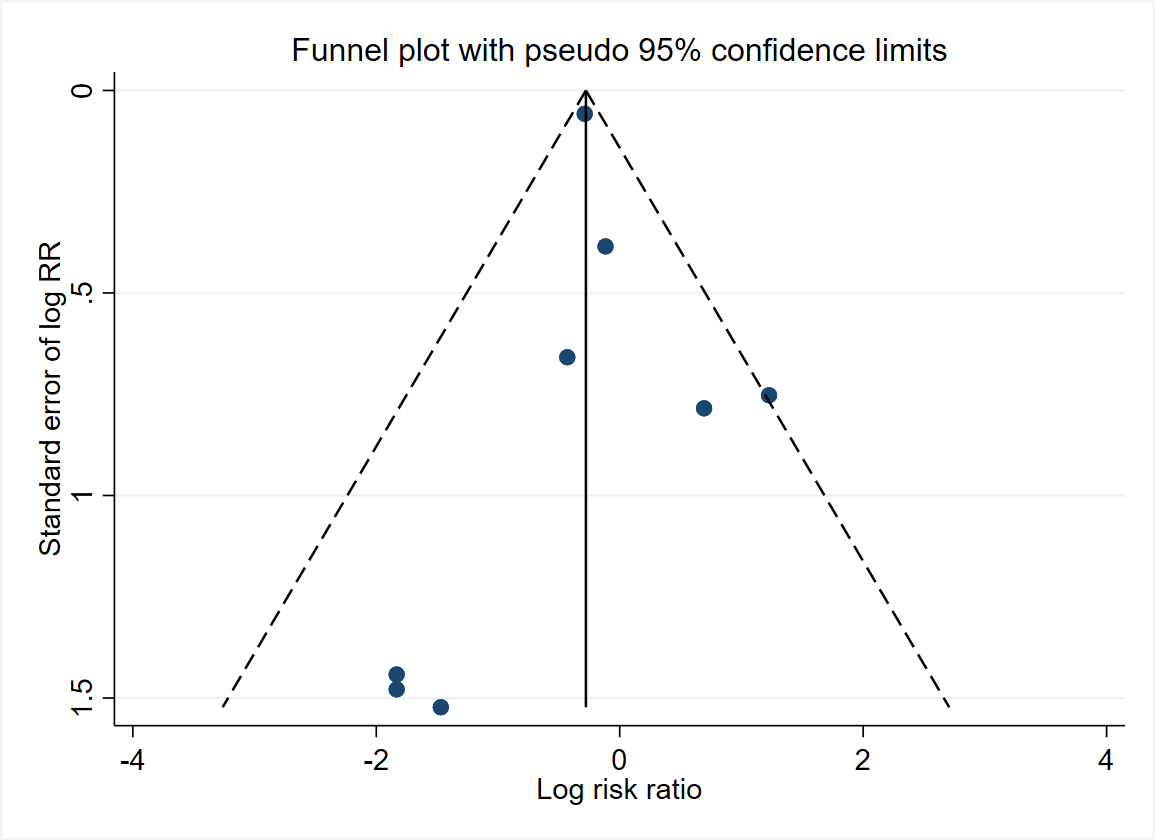

Supplement: Supplemental Information 6 [file peerj-13-20045-s006.tif]

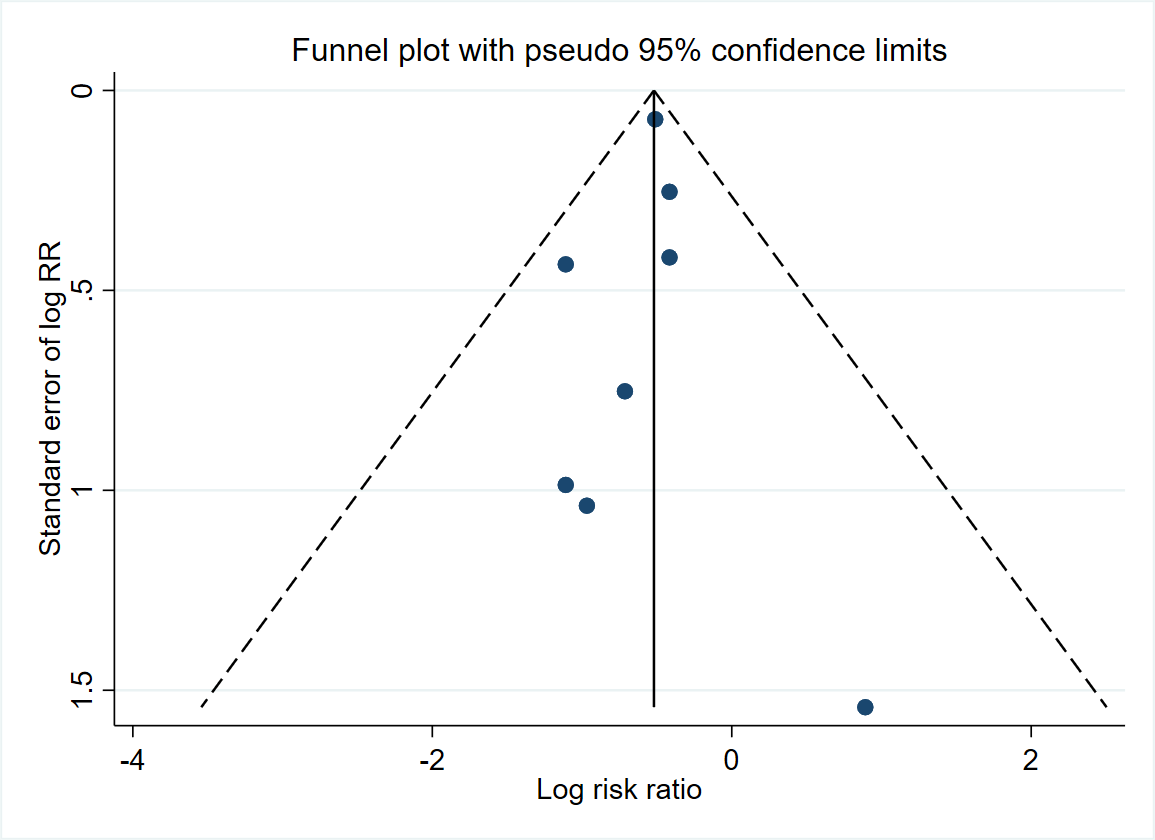

Supplement: Supplemental Information 7 [file peerj-13-20045-s007.tif]

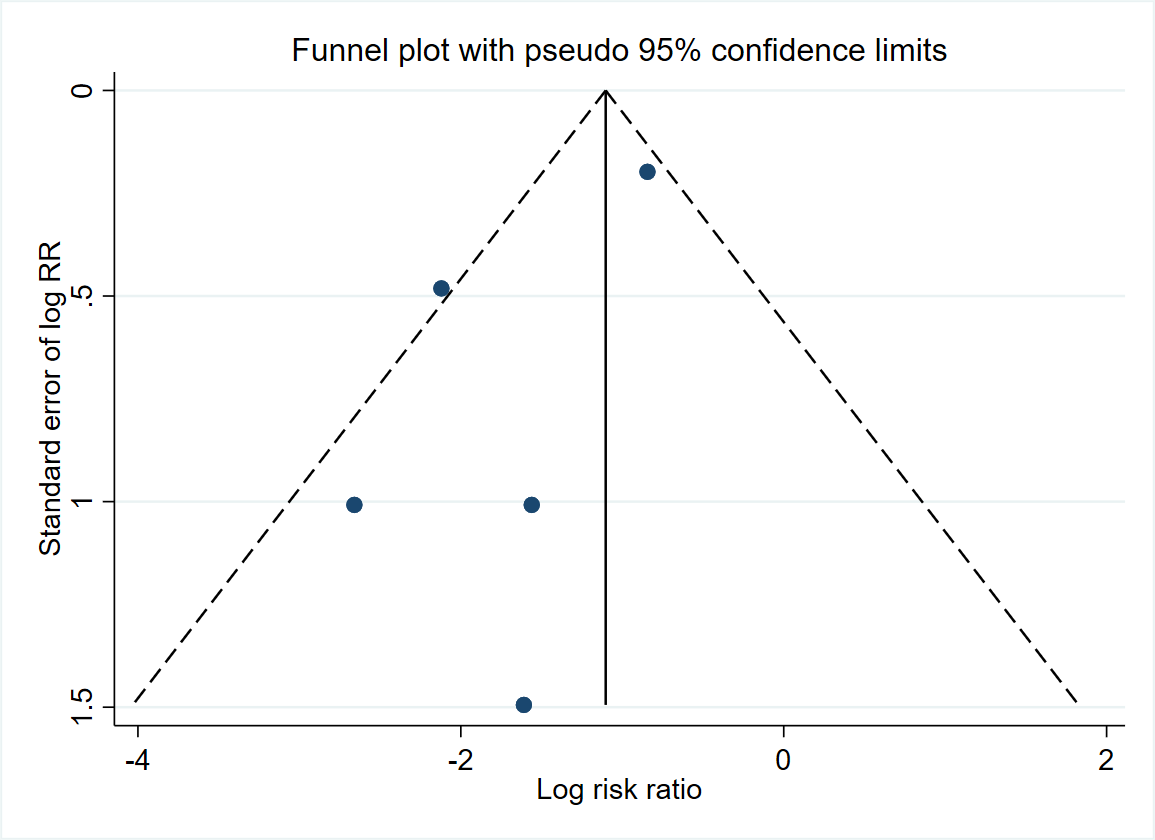

Supplement: Supplemental Information 8 [file peerj-13-20045-s008.tif]

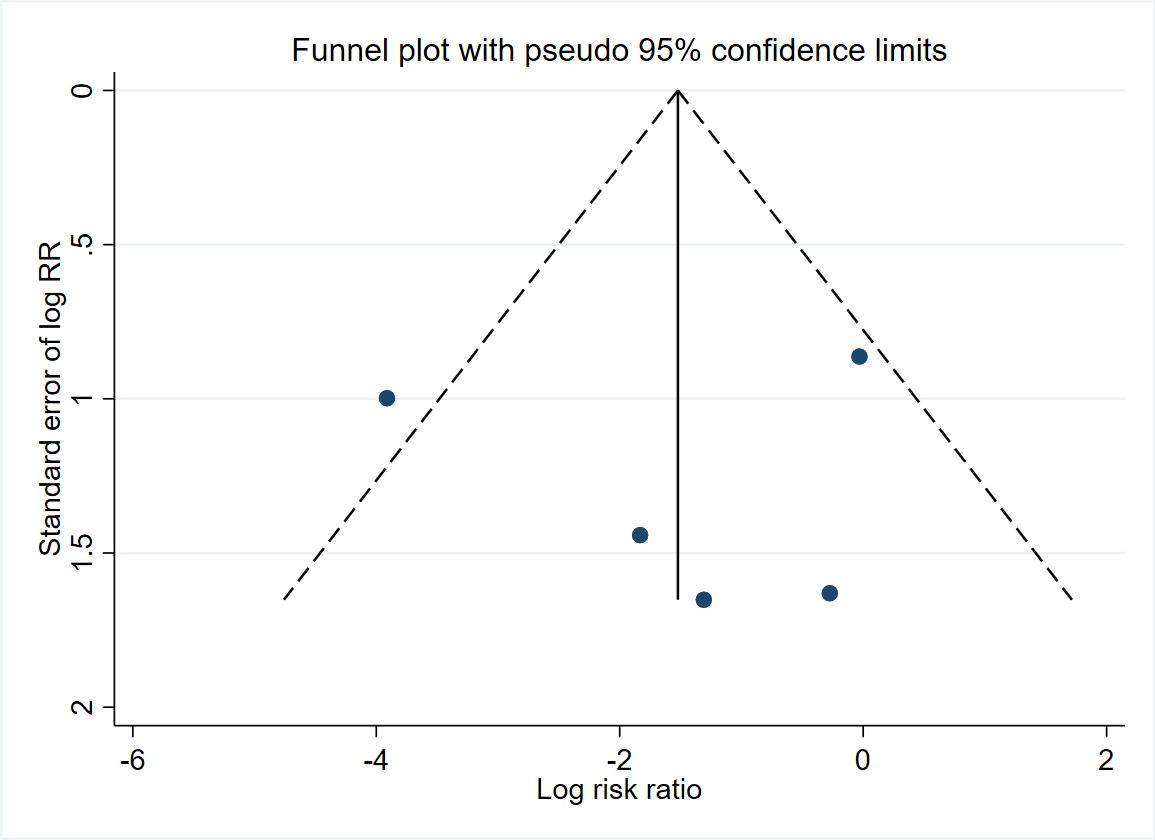

Supplement: Supplemental Information 9 [file peerj-13-20045-s009.tif]

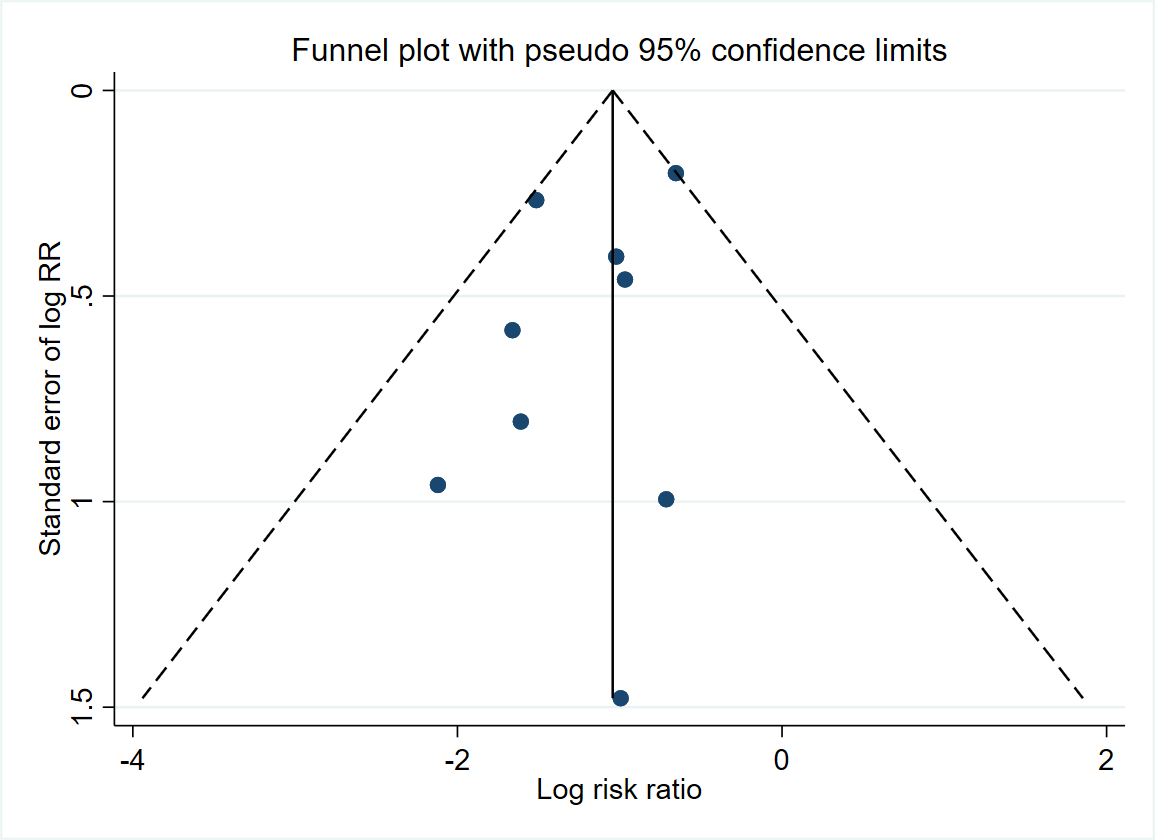

Supplement: Supplemental Information 10 [file peerj-13-20045-s010.tif]

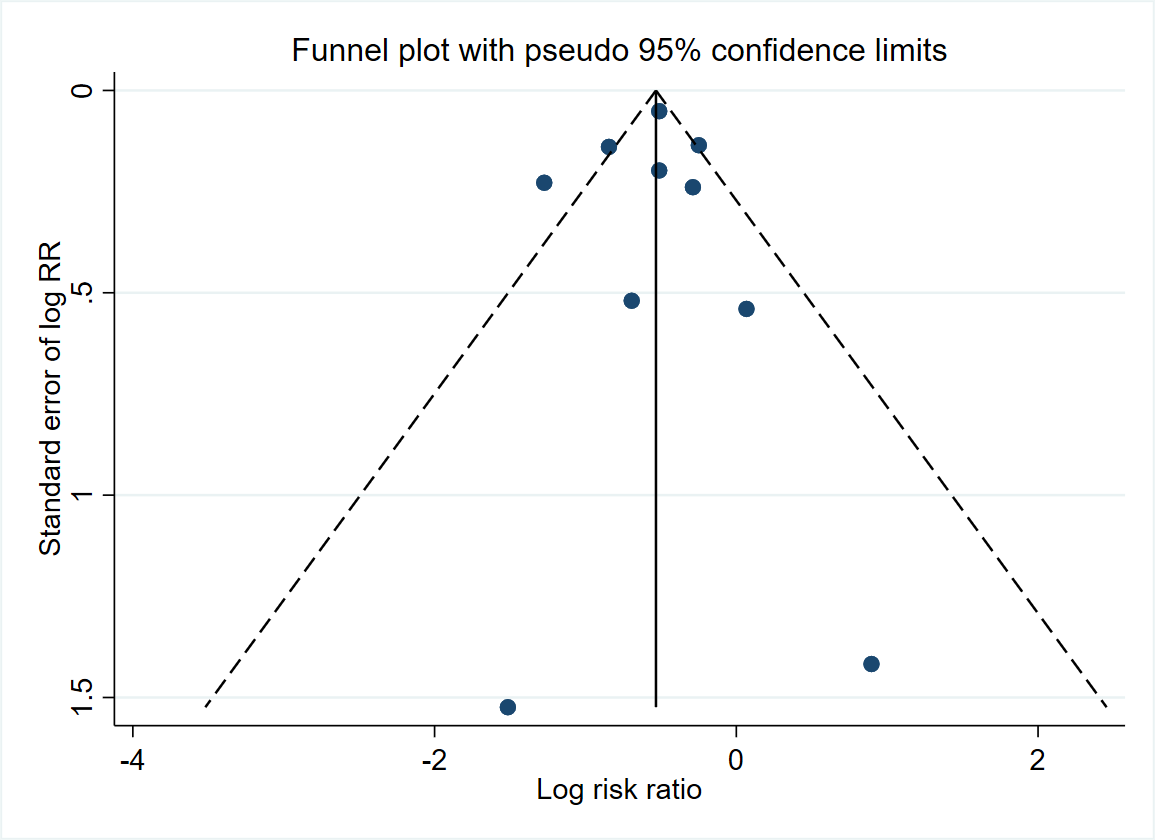

Supplement: Supplemental Information 11 [file peerj-13-20045-s011.tif]

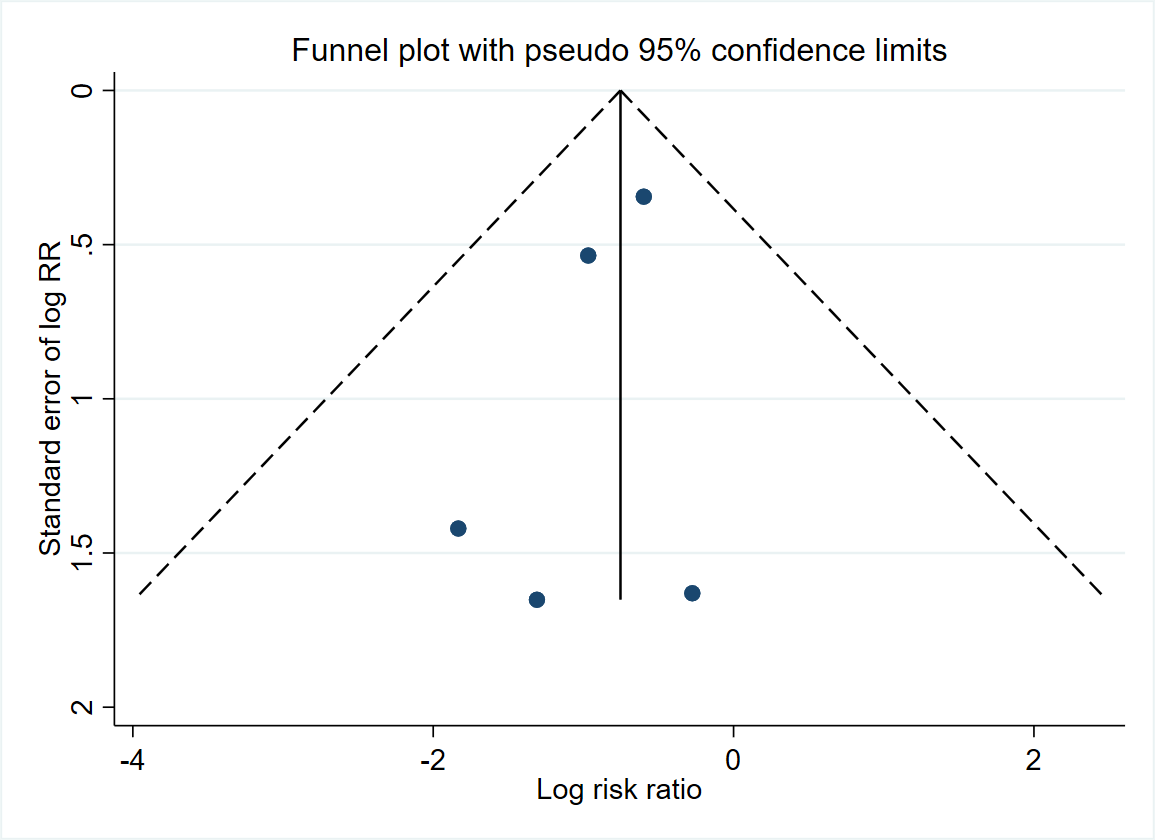

Supplement: Supplemental Information 12 [file peerj-13-20045-s012.tif]

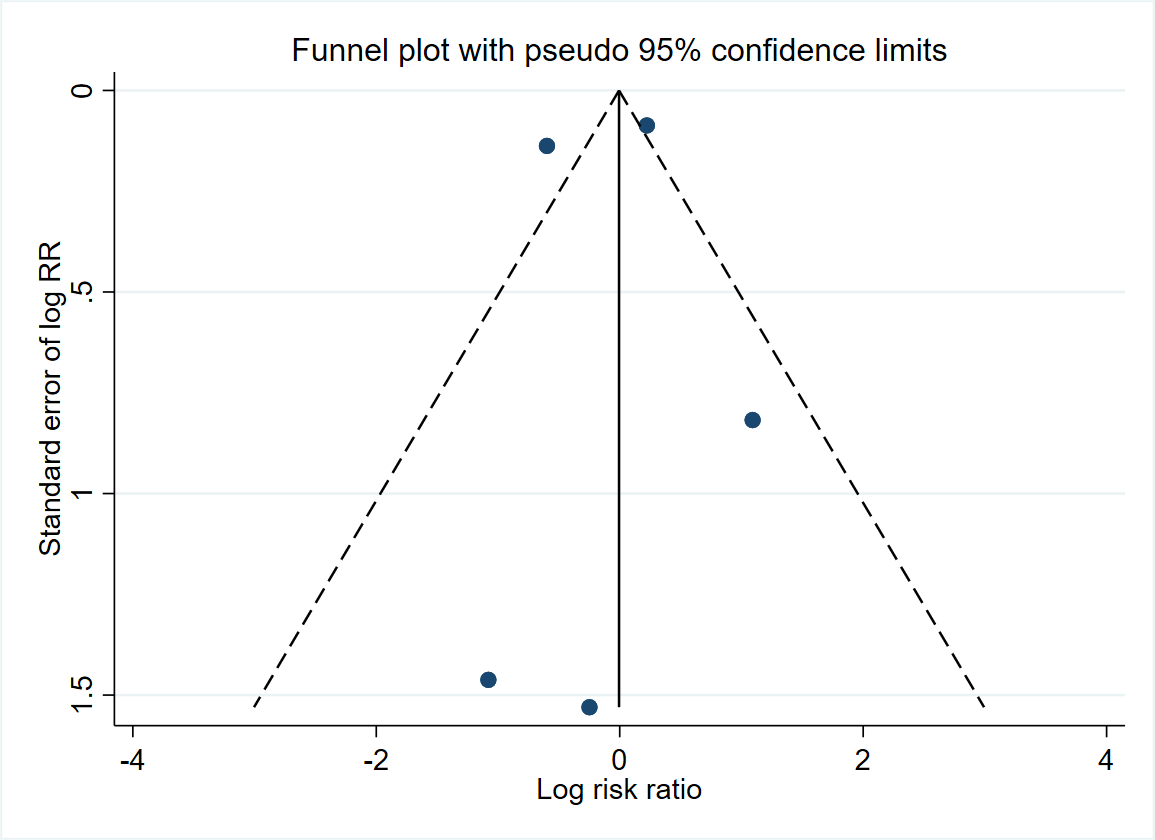

Supplement: Supplemental Information 13 [file peerj-13-20045-s013.tif]

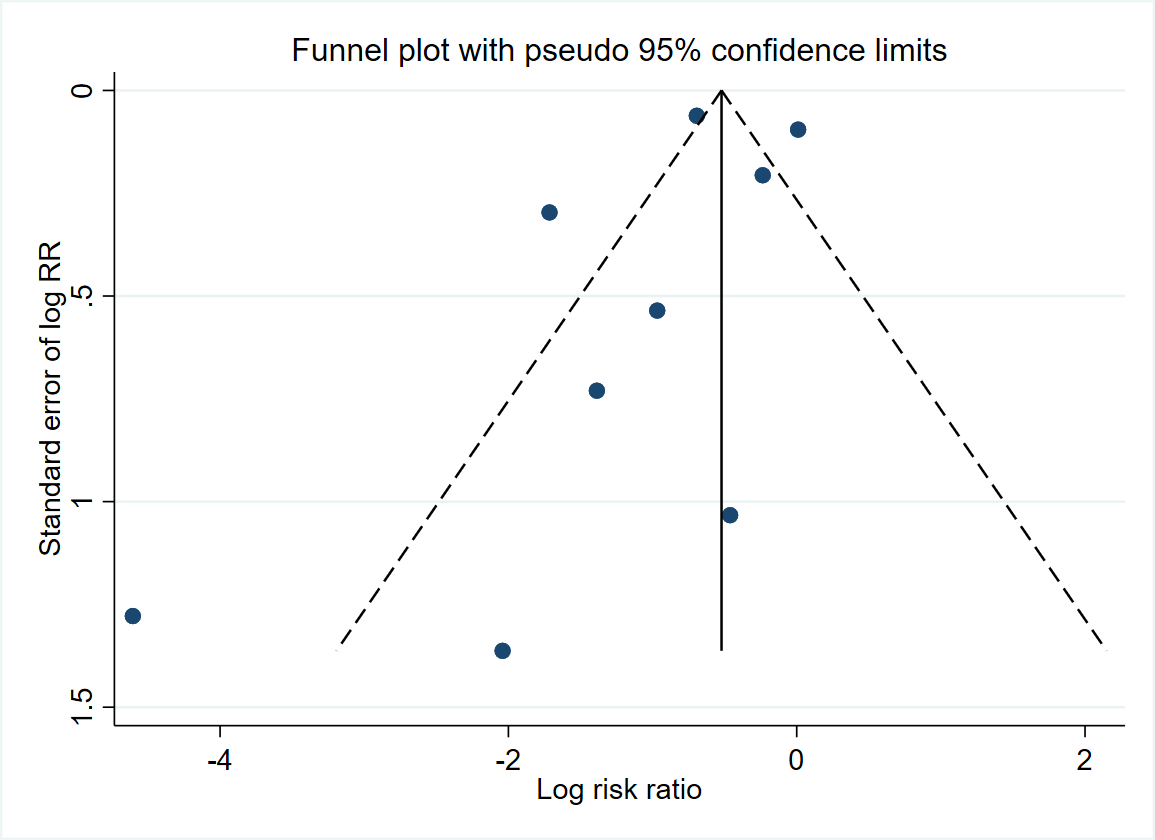

Supplement: Supplemental Information 14 [file peerj-13-20045-s014.tif]

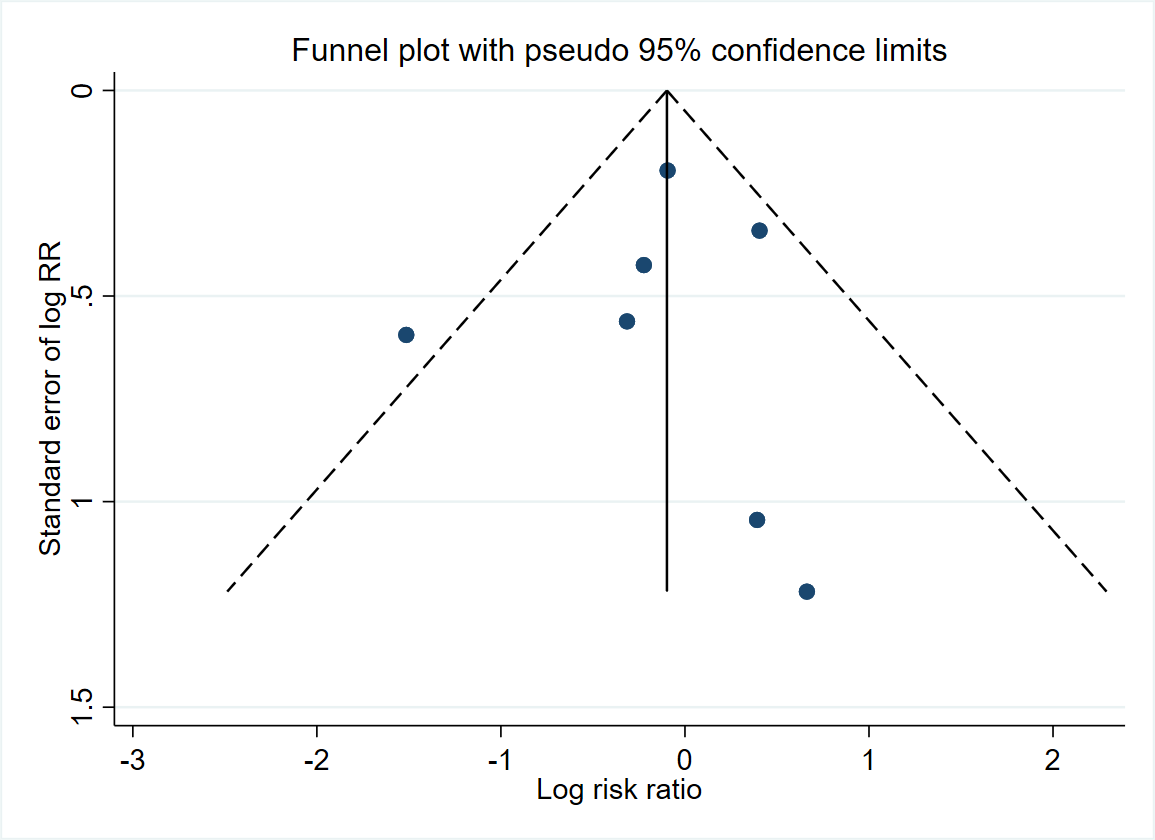

Supplement: Supplemental Information 15 [file peerj-13-20045-s015.tif]

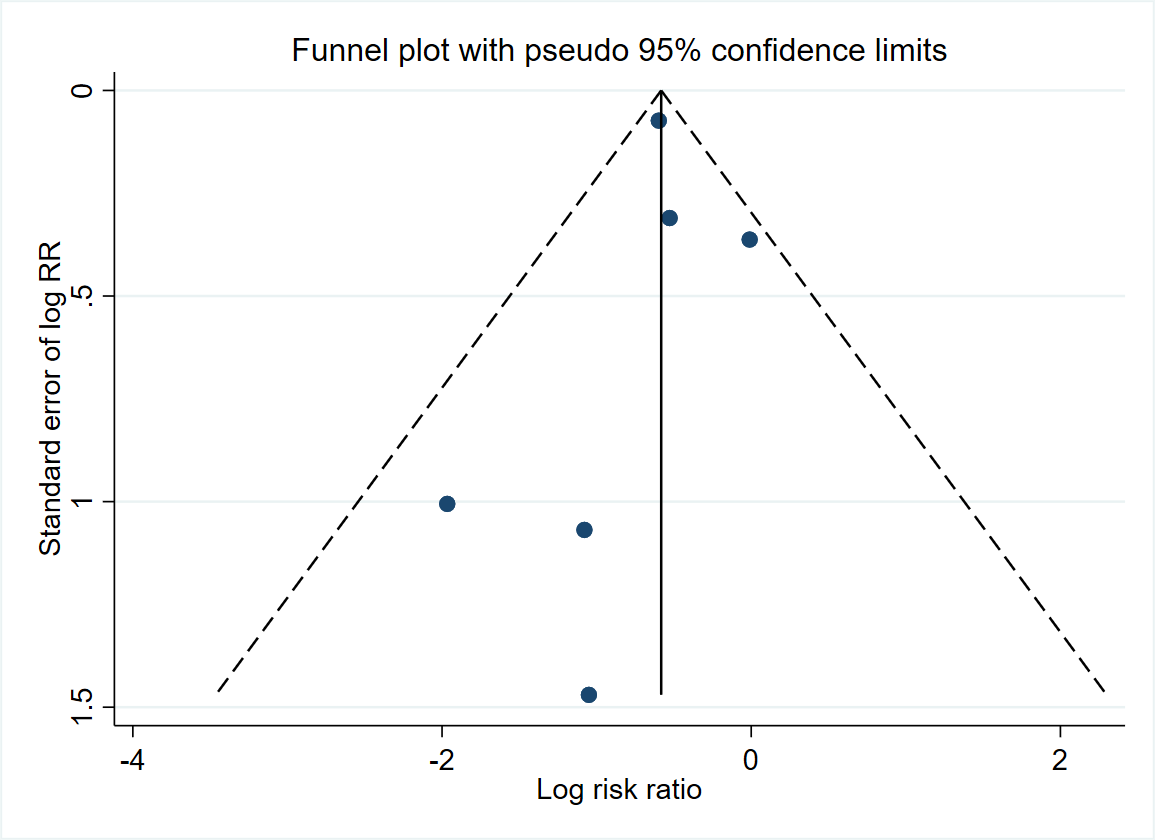

Supplement: Supplemental Information 16 [file peerj-13-20045-s016.tif]

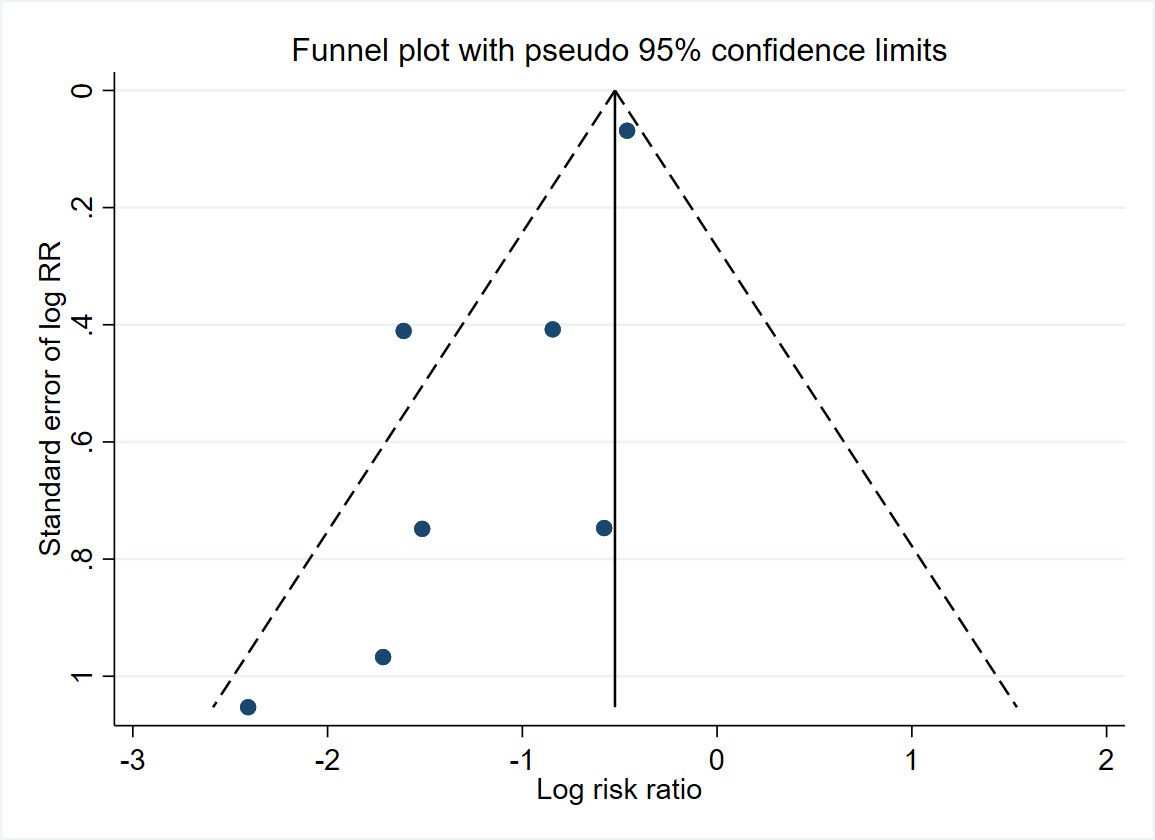

Supplement: Supplemental Information 17 [file peerj-13-20045-s017.tif]

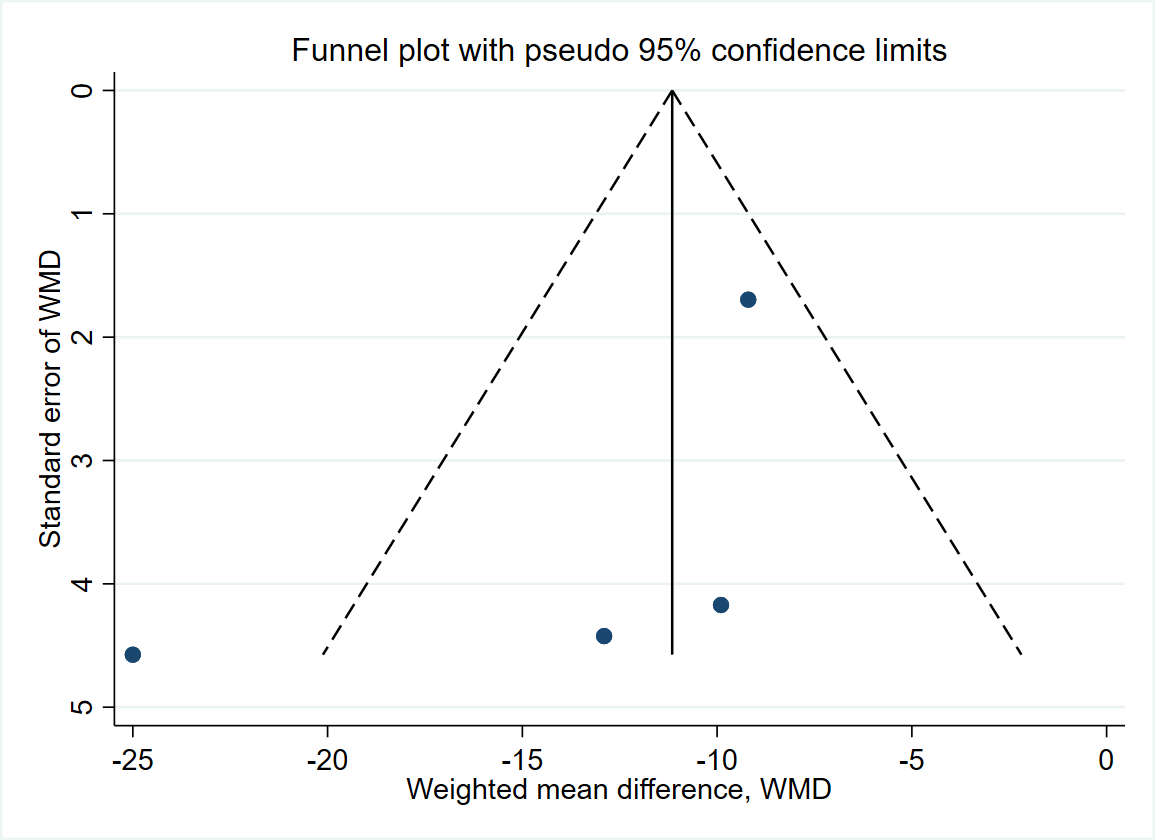

Supplement: Supplemental Information 18 [file peerj-13-20045-s018.tif]

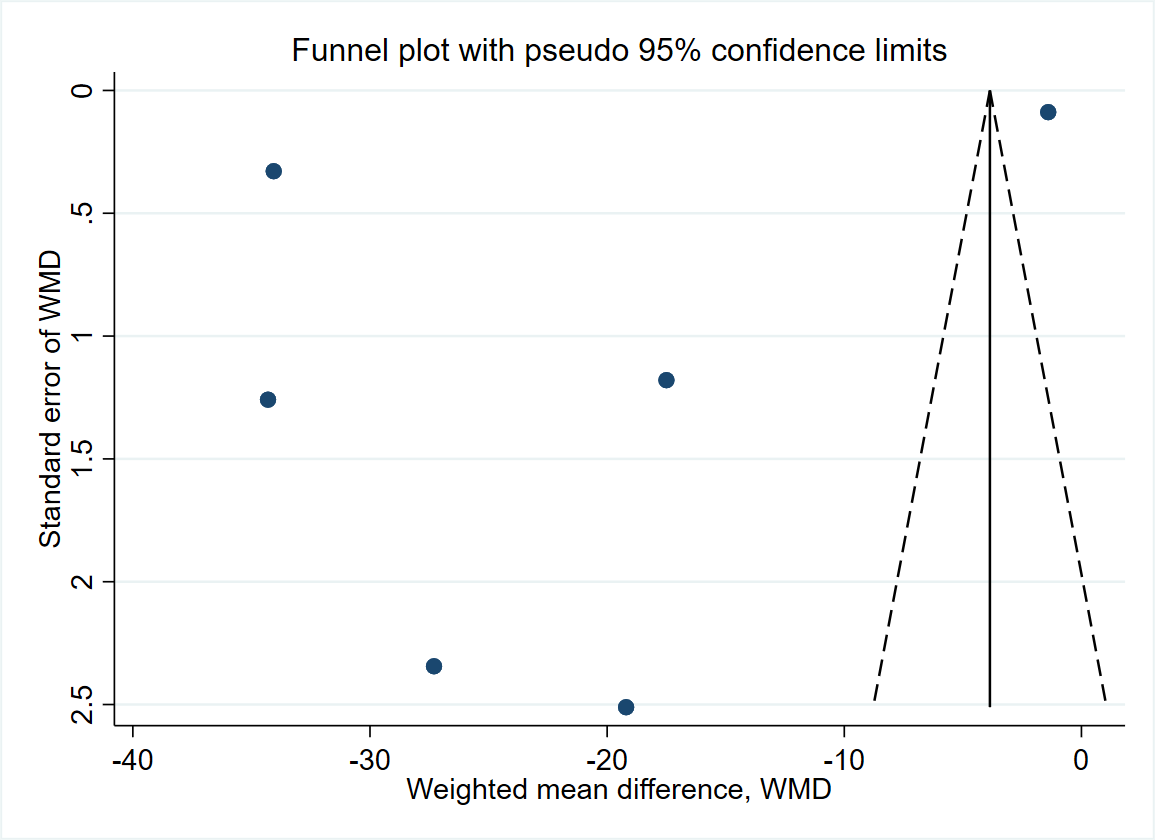

Supplement: Supplemental Information 19 [file peerj-13-20045-s019.tif]
