# Supplementary material for: Safety of outpatient vs. inpatient anterior cervical discectomy and fusion: a systematic review and meta-analysis
Source: PeerJ. 2025 Sep 22;13:e20045. doi: 10.7717/peerj.20045 (PMC12462687; doi:10.7717/peerj.20045)
Supplement: Supplemental Information 20 [file peerj-13-20045-s020.docx]

## **Supplementary Table 1. Search terms used in different databases**

| **Database** | **Search Strategy** |
| --- | --- |
| PubMed | (("anterior cervical discectomy and fusion"[MeSH Terms] OR "ACDF"[Title/Abstract] OR "cervical discectomy"[Title/Abstract])  AND ("outpatient"[Title/Abstract] OR "ambulatory surgery"[Title/Abstract] OR "same-day surgery"[Title/Abstract])  AND ("inpatient"[Title/Abstract] OR "hospital stay"[Title/Abstract])  AND ("complications"[MeSH Terms] OR "safety"[Title/Abstract] OR "mortality"[Title/Abstract] OR "readmission"[Title/Abstract] OR "reoperation"[Title/Abstract]))  AND ("humans"[MeSH Terms])  AND ("english"[Language])  AND ("2000/01/01"[Date - Publication] : "2024/12/31"[Date - Publication]) |
| Embase | (('anterior cervical discectomy and fusion'/exp OR 'ACDF':ti,ab OR 'cervical discectomy':ti,ab)  AND ('outpatient':ti,ab OR 'ambulatory surgery':ti,ab OR 'same-day surgery':ti,ab)  AND ('inpatient':ti,ab OR 'hospital stay':ti,ab)  AND ('complications'/exp OR 'safety':ti,ab OR 'mortality':ti,ab OR 'readmission':ti,ab OR 'reoperation':ti,ab))  AND [humans]/lim  AND [english]/lim  AND [2000-2024]/py |
| Scopus | (TITLE-ABS-KEY("anterior cervical discectomy and fusion" OR "ACDF" OR "cervical discectomy")  AND TITLE-ABS-KEY("outpatient" OR "ambulatory surgery" OR "same-day surgery")  AND TITLE-ABS-KEY("inpatient" OR "hospital stay")  AND TITLE-ABS-KEY("complications" OR "safety" OR "mortality" OR "readmission" OR "reoperation"))  AND (LIMIT-TO(LANGUAGE, "English"))  AND (LIMIT-TO(DOCTYPE, "ar"))  AND (PUBYEAR > 1999 AND PUBYEAR < 2025) |
