# Supplementary material for: Safety of outpatient vs. inpatient anterior cervical discectomy and fusion: a systematic review and meta-analysis
Source: PeerJ. 2025 Sep 22;13:e20045. doi: 10.7717/peerj.20045 (PMC12462687; doi:10.7717/peerj.20045)
Supplement: Supplemental Information 21 [file peerj-13-20045-s021.docx]

## **Supplementary Table 2. Newcastle Ottawa Scale (NOS) Based Quality Assessment of Included Cohort Studies**

| Study Identifier | 1. Representativeness of the exposed cohort | 2. Selection of the non-exposed cohort | 3. Ascertainment of exposure | 4. Outcome not present at start of study | 5. Comparability of cohorts | 6. Assessment of outcome | 7. Follow-up long enough | 8. Adequacy of follow-up | Total Score |
| --- | --- | --- | --- | --- | --- | --- | --- | --- | --- |
| Tani S et al (2023) | 1 | 1 | 1 | 1 | 2 | 1 | 1 | 0 | 7 |
| Boddapati V et al (2021) | 1 | 1 | 1 | 1 | 2 | 1 | 1 | 1 | 8 |
| Kamalapathy PN et al (2021) | 1 | 1 | 1 | 1 | 2 | 1 | 1 | 0 | 7 |
| Lee R et al (2020) | 1 | 1 | 1 | 1 | 2 | 1 | 1 | 1 | 8 |
| Vaishnav A et al_1 (2019) | 1 | 1 | 1 | 1 | 2 | 1 | 1 | 1 | 8 |
| Shenoy K et al (2019) | 1 | 1 | 1 | 1 | 1 | 1 | 0 | 0 | 6 |
| Patel DV et al (2019) | 1 | 1 | 1 | 1 | 2 | 1 | 1 | 0 | 7 |
| Vaishnav A et al _2 (2019) | 1 | 1 | 1 | 1 | 2 | 1 | 1 | 0 | 7 |
| Khalid SI et al (2019) | 1 | 1 | 1 | 1 | 2 | 1 | 1 | 1 | 8 |
| Purger DA et al (2019) | 1 | 1 | 1 | 1 | 2 | 1 | 1 | 0 | 7 |
| Khanna R et al (2018) | 1 | 1 | 1 | 1 | 2 | 1 | 1 | 1 | 8 |
| Mullins J et al (2018) | 1 | 1 | 1 | 1 | 1 | 1 | 0 | 0 | 6 |
| Arshi A et al (2018) | 1 | 1 | 1 | 1 | 2 | 1 | 1 | 1 | 8 |
| Purger DA et al (2018) | 1 | 1 | 1 | 1 | 2 | 1 | 1 | 0 | 7 |
| Fu MC et al (2017) | 1 | 1 | 1 | 1 | 2 | 1 | 1 | 1 | 8 |
| McClelland S et al (2017) | 1 | 1 | 1 | 1 | 1 | 1 | 0 | 0 | 6 |
| Adamson T et al (2016) | 1 | 1 | 1 | 1 | 2 | 1 | 1 | 0 | 7 |
| McGirt MJ et al (2015) | 1 | 1 | 1 | 1 | 2 | 1 | 1 | 1 | 8 |
| Martin CT et al (2014) | 1 | 1 | 1 | 1 | 2 | 1 | 1 | 1 | 8 |
| Liu JT et al (2009) | 1 | 1 | 1 | 1 | 1 | 1 | 0 | 0 | 6 |
| Stieber JR et al (2005) | 1 | 1 | 1 | 1 | 1 | 1 | 0 | 0 | 6 |
